# Evaluation of an App-based brief Cognitive Behavioral Therapy for individuals with Nonsuicidal Self-injury

**DOI:** 10.1192/j.eurpsy.2024.1146

**Published:** 2024-08-27

**Authors:** S. Kang, S. M. Zoh, J.-W. Hur

**Affiliations:** Department of Psychology, Korea University, Seoul, Korea, Republic Of

## Abstract

**Introduction:**

Nonsuicidal self-injury (NSSI), the deliberate and direct destruction of one’s own body tissue without suicidal intent, has represented a significant public health concern among adolescents and young adults worldwide, yet they have limited access to evidence-based interventions. App-based digital therapy, with its advantages of high cost-effectiveness, accessibility, and user receptivity, could be an effective intervention for NSSI. We expected that the use of an app-based brief cognitive-behavioral therapy (CBT) would improve depressive symptoms and emotion dysregulation, the most prevalent symptoms among individuals with NSSI.

**Objectives:**

This study aimed to evaluate the efficacy of a 3-week app-based brief CBT program focusing on cognitive distortion correction for individuals with NSSI.

**Methods:**

A total of 34 participants who engaged in NSSI were included in the final analysis, with 18 individuals assigned to the ‘app group’ and 16 to the ‘waitlist group.’ The brief CBT program consisted of three quizzes designed to prompt the users to identify cognitive distortions embedded in a series of short scenarios, develop more realistic perspectives, and imagine advising to significant others. The app group was instructed to complete three quizzes per day for three weeks, while the waitlist group received no intervention.

**Results:**

Baseline and follow-up assessments of depression and emotion regulation were conducted. After the 3-week program, the app group showed a significant reduction in depressive symptoms (*F* = 8.30, *P* = .007) compared to the waitlist group. There was no group difference regarding emotion regulation.

**Conclusions:**

Depression is a prominent symptom in individuals with NSSI. Our findings suggest that an app-based brief CBT intervention targeting cognitive distortions can effectively alleviate depression in individuals with NSSI. The results also highlight the need for digital interventions that are tailored and designed to improve emotion regulation in this population.

**Disclosure of Interest:**

None Declared

